# “I’m always going to be tired”: a qualitative exploration of adolescents’ experiences of fatigue in depression

**DOI:** 10.1007/s00787-023-02243-3

**Published:** 2023-06-10

**Authors:** Nina Higson-Sweeney, Kate Cooper, Barnaby D. Dunn, Maria E. Loades

**Affiliations:** 1 Department of Psychology, University of Bath, Claverton Down, Bath, BA2 7AY UK; 2 Mood Disorders Centre, University of Exeter, Exeter, EX4 4QG UK

**Keywords:** Adolescence, Depression, Fatigue, Lived experience, Qualitative

## Abstract

Adolescent depression is a prevalent and disabling condition, but current psychological treatments are only moderately effective. One way to enhance outcomes is to further our understanding of adolescent depression and improve our capacity to target the most frequently reported and problematic symptoms. A common but often neglected symptom of depression is fatigue, which is associated with considerable impairment and has the potential to interfere with adolescents’ engagement in psychological therapies. Despite this, the experience of fatigue in adolescent depression and how we target it in treatment is currently poorly understood. Therefore, we aimed to explore adolescents’ experiences and understandings of fatigue in depression, recruiting from clinical and community settings. Semi-structured interviews were conducted with 19 UK-based adolescents aged 14–18 years old with elevated symptoms of depression. Using reflexive thematic analysis, three themes were generated. *Fatigue is a complex concept* explored adolescents’ understanding of fatigue as a dynamic, multifaceted symptom which had mental and physical components. *Trapped in a cycle of fatigue* considered the complex and reciprocal relationship between fatigue and other depressive symptoms, and the subsequent impact of limited energy on engagement with everyday activities. Finally, *stigma as a barrier to help-seeking* highlighted how adolescents were reluctant to seek help due to experienced stigma and the perception that fatigue was not a serious enough symptom. Findings from this study suggest that fatigue should be viewed as a psychological as well as somatic symptom of depression, with implications regarding the identification and treatment of fatigue in depression in routine clinical practice.

**Supplementary Information:**

The online version contains supplementary material available at 10.1007/s00787-023-02243-3.

## Introduction

Major Depressive Disorder (MDD), also known as depression, is one of the leading causes of health-related disability in adolescents worldwide [1]. Depression has a common onset in adolescence, with approximately 20% of adolescents experiencing a depressive episode before the age of 18 [2]. The estimated worldwide 1-year prevalence of diagnosed MDD in adolescents is 8%, increasing to 19% over the lifetime [3]. Adolescent depression is a serious public health concern, as it has repeatedly been associated with negative long-term outcomes, such as an increased likelihood of experiencing recurrent depressive episodes in adulthood [4, 5], reduced social functioning, educational underachievement [6], and heightened risk of substance misuse and suicidal and self-harming behaviours [7]. Therefore, it is important that adolescent depression is identified and addressed early and effectively. Yet, current psychological treatments are moderately effective for this population at best [8].

Research indicates that adolescents may endorse different depressive symptoms to adults, such as irritability or sleep disturbances [9, 10]. This is recognised by the explicit inclusion of irritability as a core symptom of depression in under 18’s in the current Diagnostic and Statistical Manual of Mental Disorders (DSM-5) [11], although treatments tend to be downward extensions of adult protocols. A potential way to improve treatments for adolescent depression is to identify which symptoms are most prevalent and problematic for this population. In turn, this information may guide how we target these symptoms.

Fatigue is common in adolescence, irrespective of depression. Defined as an extreme state of physical and/or mental exhaustion following normal activities [12], fatigue affects approximately 21% to 38% of adolescents [13]. In the long term, fatigue has been linked with poor psychosocial outcomes such as school absenteeism, impaired social development, and a reduction in leisure activities [14], all of which affect the normal trajectory of adolescent development.

Fatigue is also a specific symptom of MDD [11], and the importance of this symptom within depression has gained increased attention in adult populations. In adults, it is recognised that up to 95% of patients will experience fatigue as a symptom of depression [15]. The presence of either fatigue or depression increases the likelihood of experiencing the other twofold [16]. Fatigue in adult depression has been repeatedly linked with greater depression severity [17], and has also been associated with greater functional impairment [15] and increased economic burden [17, 18]. In comparison to other symptoms of depression, such as low mood, fatigue responds less well to treatments like antidepressants [19] and is often experienced as a residual symptom after otherwise successful treatment, affecting 20% to 38% of adult patients in remission from depression [20, 21]. As a residual symptom of depression, fatigue in adults is predictive of future relapse and greater psychosocial impairment, suicidality, and chronicity [22].

Although both fatigue and depression are common and disabling for adolescents, much less is known about the relationship between the two in this population. Studies investigating the prevalence of MDD symptoms in adolescents indicate that 43% to 73.3% of adolescents with depression report fatigue as a symptom [23, 24], and that it is the most commonly endorsed somatic symptom in this population [25]. Fatigue and depression in adolescents have been found to highly correlate and covary over time [26], and depressed adolescents may experience more impairing and distressing fatigue than their healthy and chronically ill counterparts [27], indicating that there might be a unique relationship between fatigue and depression. Experiencing fatigue may also interfere with psychological treatments for depression, limiting an adolescent’s ability to engage with therapy and utilise the techniques learnt [28, 29]. Therefore, we need to better understand fatigue as a symptom of depression for adolescents.

Qualitative approaches allow for the exploration of phenomena in great depth, directly from individuals with lived experience [30]. Existing qualitative studies have typically focused either on the experience of adolescent depression more broadly [31–33] or on the impact of specific psychological symptoms, like anhedonia [34]. Despite this, many of these studies have mentioned the impact of fatigue. For example, Watson et al. [34] explored how low energy impacted adolescents’ ability to engage in pleasurable activities, and Midgley et al. [31] explored the negative impact of fatigue on education, and its contribution to depressed adolescents’ “bleak view of everything” (pg. 274). This highlights the potential interactions fatigue may have with other symptoms of depression, which could have significant implications. Qualitative studies that have explored fatigue in adolescent depression have focused on the symptom in the context of Cognitive Behavioural Therapy (CBT) [29] and in how clinicians address fatigue and sleep difficulties in routine mental health practice [35]. This means that there is still a gap in the experiential account of fatigue in adolescent depression, and the need for further research.

Therefore, the current study aimed to qualitatively explore adolescent’s experiences of fatigue within depression. By doing this, we hoped to increase our understanding of how fatigue is experienced, described, and understood by adolescents with elevated symptoms of depression in the UK, how fatigue interacts with other symptoms of depression, and the impact it has on their lives.

## Method

### Participants

Adolescents were eligible to take part if they were 11–18 years old, living in the UK, fluent in English, and experiencing elevated symptoms of depression (score of ≥ 8 on the Short Mood and Feelings Questionnaire [SMFQ; 36]). As Child and Adolescent Mental Health Services (CAMHS) are the main route for adolescents in the UK to seek National Health Service (NHS) mental health support, participants were recruited through CAMHS in South-West England, with clinicians excluding adolescents who were at high risk of self-harm or suicide. As many adolescents with depression do not have access to CAMHS services [37], we also recruited through adverts shared on social media (Facebook, Instagram) and recruitment websites like MQ Mental Health.

We anticipated we would need to recruit 15–30 adolescents, based on sample sizes from previous qualitative studies with similar research aims [32, 34]. However, recruitment was governed by the concept of information power [38], with recruitment ending once the research team determined that enough data of sufficient quality and depth had been collected to answer the research questions.

### Procedure and materials

The study received ethical approval from the NHS Research Ethics Committee (IRAS ID: 302262) and the University of Bath’s Psychology Research Ethics Committee (21-242).

Adolescents attending CAMHS services who were potentially eligible to take part were given a copy of the study advert by their clinician during a routine appointment and provided verbal consent for researchers to contact them with further information. Study adverts shared on social media and MQ Mental Health included a link to an online information sheet, which invited interested adolescents to email the research team. The study was open to recruitment from November 2021 to April 2022, meaning interviews partially took place within the context of the COVID-19 pandemic. Interested adolescents were asked by the research team to complete the SMFQ [36] to assess depressive symptom severity and as a screen for eligibility for study participation (i.e., elevated depression symptoms). The SMFQ is a validated and reliable 13-item self-report questionnaire which asks individuals to rate each item on a three-point Likert scale ranging from 0, meaning ‘not true’, to 2, meaning ‘true’. To further contextualise the sample and ensure the presence of problematic fatigue, adolescents also completed the Chalder Fatigue Scale (CFQ) [39], an 11-item questionnaire designed to identify the presence or absence of fatigue. The scale was scored bimodally, with two responses representing 0 (‘less than usual’ and ‘no more than usual’) and two responses representing 1 (‘more than usual’ and ‘much more than usual’). This resulted in a score from 0 to 11, with a score of ≥ 4 indicating the presence of marked fatigue.

Informed written consent was obtained from all participants, and from parents/guardians when participants were under 16 years old. Participants then took part in a semi-structured interview with the first author (NH-S), which was either online via Microsoft Teams or over the telephone. Participants were given the option of having parents/guardians in attendance, but none opted for this. Interviews were audio-recorded and lasted an average of 38 min (range 21–52 min).

Interviews followed a flexible topic guide, developed by the research team with input from a Young Person’s Advisory Group, who provided consultation across the project. The topic guide comprised a series of open-ended questions grouped into three related areas: (1) Knowledge, understanding and descriptions of fatigue; (2) Personal experiences of fatigue; and (3) Personal experiences of depression and involvement of fatigue. Questions were accompanied by prompts, which ranged from specific (e.g., “What does hearing the word ‘fatigue’ make you think of?”) to general (e.g., “How did that make you feel?”). The topic guide was revisited and refined throughout the data collection process to ensure accuracy and that the questions were appropriate for the participants (see online supplementary materials).

Due to the sensitive nature of the research and recruitment of adolescent participants with mental health needs, a comprehensive risk management plan was developed for the study, including procedures for identifying and addressing risk of self-harm or suicide or risk to others. This was identified prior, during, and after interviews, and involved communication of pertinent risk issues to parents/guardians, CAMHS clinicians, and general practitioners (full study protocol can be found on the Open Science Framework: https://osf.io/wdnvg).

After the interview, all participants received a £10 gift voucher for their participation. Interviews were then transcribed verbatim by NH-S and research assistants, at which point any identifying information was removed, and pseudonyms were assigned.

### Data analysis

Qualitative data were analyzed using reflexive thematic analysis (RTA), which is a method of systematically identifying and organising patterns of meaning across a dataset [40, 41]. This approach was chosen as it is well-suited to research seeking to make sense of shared meanings and experiences, particularly in under-explored areas [30, 40]. RTA is also a widely used approach, with established parameters to facilitate high quality, robust analyses [41]. An inductive approach was taken, meaning the analysis was data-driven and stuck closely to participants’ accounts.

RTA is theoretically flexible, meaning it does not have a specific epistemological or ontological framework [41]. For the current study, the researchers worked within an experiential framework, approached from a critical realist, contextualist perspective. An experiential framework “prioritises the examination of how a given phenomenon may be experienced by the participant” (p.1396), understanding that although any thoughts, feelings and experiences are subjective, they reflect an inner, personal state held by the participant [42]. A critical realist approach assumes that whilst the world is knowable, there is no one objective reality [30]. Combined with a contextualist approach, what a participant understands as ‘reality’, and any knowledge generated of this, is the product of the specific context they are living in [30]. In this way there are multiple realities and knowledge is always provisional. This approach understands that whilst we are unable to gain direct access to individuals’ realities, research can allow us partial insight, and from this we can make observations that may be transferable to other, similar contexts [43].

The researchers actively considered how their own values, experiences, beliefs, and identities shaped the study [36], including knowledge and experience of working in CAMHS (KC, MEL), conducting research into child and adolescent mental health (NH-S, KC, BDD, MEL), and personal experiences with mental health (NH-S; see [44]). This was achieved through group supervisory meetings for the duration of the project and reflexive diaries (see online supplementary materials for reflexivity statement).

Qualitative analysis was guided by Braun and Clarke’s [41] six-phase process. In phase 1, NH-S became familiar with the data by reading and re-reading the transcripts and creating mind-maps depicting each participants’ experience. During phase 2, NH-S conducted line-by-line coding, focusing on data most salient with the research questions. Coding was done at a semantic and latent level and was inductive, meaning codes were driven by the data and stuck closely to participants’ accounts. Coding was a recursive process, involving multiple read-throughs and comparisons within and between transcripts. Input was sought during this phase from KC and MEL. Phase 3 involved generation of initial themes, where NH-S considered the dataset as a whole and began to subsume codes into potential themes and subthemes. In phases 4 and 5, KC and MEL aided NH-S in reviewing and refining codes and tentative themes, before developing names and definitions. Phase 6 involved the final write-up, with feedback from the whole author team. NVivo software was used to conduct the analysis.

Questionnaire data from the SMFQ and CFQ were used descriptively to characterise the sample.

## Results

### Participant characteristics

Of the 27 adolescents who were screened, 24 were eligible to take part. Of those eligible, 22 gave consent or assent but 3 later withdrew, resulting in the final sample of 19 adolescents. 15 participants were recruited through social media and MQ Mental Health, and 4 were recruited through CAMHS. Participants were aged 14–18 years old (*M* = 16.16, SD = 1.01), with 11 participants identifying as female, 5 as male, and 3 as non-binary. One participant also identified as transgender. Eleven participants (57.89%) were White British, with 3 participants who were White Other, 2 Pakistani, 1 White Irish, 1 African, and 1 Mixed White and Asian. Mean score for depression severity on the SMFQ was 16.89, and mean score for fatigue severity on the CFQ was 8.56 (see Table 1 for participant characteristics).

**Table 1:** Participant demographics and clinical characteristics

Pseudonym	Age^a^	Depression severity—SMFQ score (/26)	Fatigue severity—CFQ score (/11)	Self-reported comorbid physical and mental health conditions	Recruitment source	Contact with CAMHS
Agata	16	23	11	Anxiety, unspecified eating disorder, EDD, CPTSD	Online	Current
Alfie	15	21	11	Anxiety	CAMHS	Current
Amina	15	13	8	Anxiety	Online	None
Ben	16	20	10	Substance abuse	CAMHS	Current
Ellis	15	13	9	Anxiety, gender dysphoria	Online	None
Finley	17	11	5	OCD	Online	Past
Fran	17	12	5	Anxiety, ASD	Online	Past
Freya	17	19	9	Anxiety	Online	None
Imani	16	11	9	Not disclosed	Online	None
Jude	17	15	11	Anxiety, anorexia nervosa, ASD, insomnia	Online	Past
Keira	16	21	9	None	CAMHS	Current
Lila	17	21	11	ASD	Online	None
Matty	17	16	9	ADHD	Online	Past
Megan	16	23	11	Anxiety, migraines, OCD, PTSD	Online	Current
Morgan	14	22	8	Anxiety, PTSD	CAMHS	Current
Olive	17	25	8	Anorexia nervosa	Online	Current
Oskar	16	9	10	None	Online	None
Saoirse	18	14	5	Asthma	Online	None
Zainab	15	12	4	None	Online	None

*ADHD* Attention Deficit Hyperactivity Disorder, *ASD* Autism Spectrum Disorder, *CAMHS* Child and Adolescent Mental Health Services, *CFQ* Chalder Fatigue Scale, *CPTSD* Complex Post-traumatic Stress Disorder, *EDD* Emotional Dysregulation Disorder, *OCD* Obsessive Compulsive Disorder, *PTSD* Post-traumatic Stress Disorder, *SMFQ* Short Mood and Feelings Questionnaire

^a^At time of interview

### Themes

**Fig. 1:**
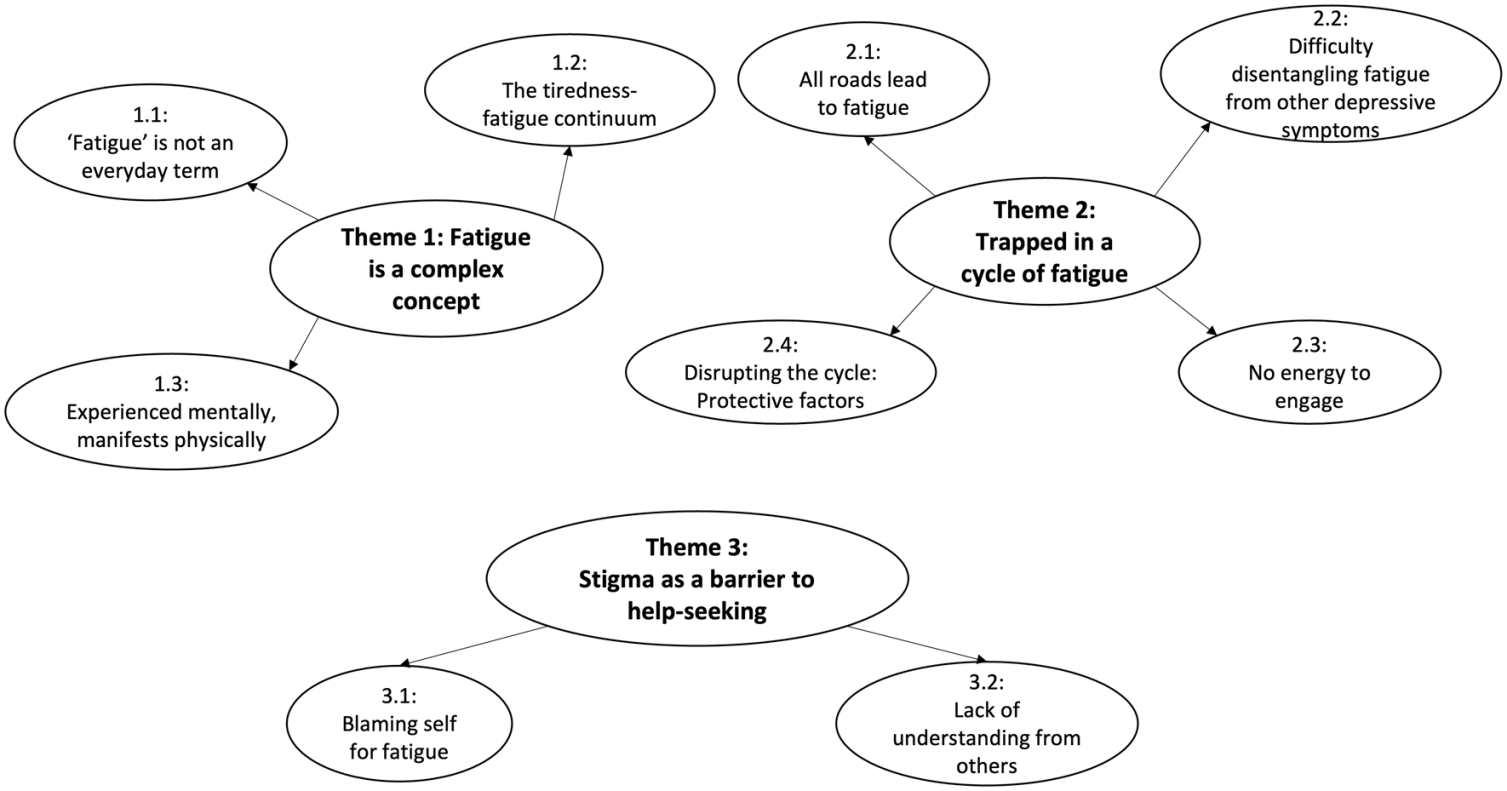
Thematic map

Adolescents’ experiences and understandings of fatigue within depression are explored through three themes: (1) Fatigue is a complex concept; (2) Trapped in a cycle of fatigue; and (3) Stigma as a barrier to help-seeking (see Fig. 1).

#### Theme 1: fatigue is a complex concept

This theme focuses on how adolescents understand and make sense of the complexity of fatigue in the context of depression and their personal experiences.

##### “It wouldn’t be my first choice”: ‘fatigue’ is not an everyday term

Adolescents used numerous words to describe the feeling of fatigue, such as “tired all the time”, “low energy”, “drained” and “exhausted”. However, when faced with the term ‘fatigue’ itself, most adolescents understood what it meant but did not always find it reflective of their experiences. Fatigue was perceived to be a *“medical”* (Zainab) term which adolescents would consider using with healthcare professionals or as a last resort, but not in day-to-day life.

“If I have like a GP visit or whatever and they’re asking in general how things are or y’know like my CAMHS meetings and whatnot I would be like ‘oh I y’know feel like really fatigued lately’ […] fatigue is not a really a word that I use around my friends.” (Olive)

##### “Everyone gets tired, y’know? But like extreme tiredness […] it’s a lot bigger”: the tiredness-fatigue continuum

Among participants, it was understood that a certain level of tiredness was a common and normal experience, particularly for teenagers; *“Pretty much every day one of my friends will be like ‘I’m tired’, ‘yeah, me too’”* (Finley). However, there seemed to be a shared understanding that at a certain point tiredness becomes problematic, which is when it is labelled as fatigue. In this way, adolescents seemed to conceptualise tiredness and fatigue as on a continuum, with fatigue as an extreme or abnormal form of tiredness.

“Tired is kind of quote unquote acceptable […] whereas fatigued to me is just like I cannot even consider doing anything […] I’d say the fatigue is more – more of an extreme end of being tired.” (Matty)

Adolescents understood this continuum of tiredness and fatigue as something that is dynamic rather than static, and recognised that fatigue could fluctuate on a weekly, daily, or even hourly basis: *“Most days I have it but like not at all points of the day like I can start my day and feel okay and then it gets like worse over the day”* (Fran). The ever-changing nature of this experience seemed to make it difficult to pinpoint exactly when tiredness becomes fatigue. However, adolescents were clear that in comparison to tiredness, fatigue is something that is overwhelming and all-encompassing, experienced persistently over a long period of time, and cannot be easily resolved by rest.

“[Fatigue is] a tiredness that isn’t just through just a kind of regular tiredness as the result of like not having enough sleep, it’s just something that feels chronic and just doesn’t go away no matter what you do.” (Lila)

##### “Even if I haven’t done something physical, it’s as if I have”: experienced mentally, manifests physically

Unprompted, many participants distinguished between two types of fatigue: mental fatigue, and physical fatigue. Physical fatigue was often discussed as something with an identifiable cause that could be easily treated.

“If you're physically tired you can just get an earlier night or just have a nap.” (Jude)

Contrastingly, mental fatigue was more strongly related to emotional overwhelm and the experience of depression.

“You can have a full night’s sleep and still be tired because you don’t necessarily have to be tired in the physical concept, you can be tired of living or tired emotionally.” (Ellis)

Although these two types of fatigue were understood to be different, adolescents recognised that they were difficult to distinguish because both can manifest physically. As Ben described: “*I find when I'm mentally drained mostly I feel physically weak um I don't know, like biologically if I am actually, you know, if my muscles are actually tired”*. This element contributed to the participants’ understanding of mental fatigue as something harder to identify and address, and thus potentially permanent, which evoked a sense of hopelessness.

“Mental is worse because it’s much harder to recharge.” (Jude)

“It just doesn’t seem to go away really. Or if it does go away, it’ll come back after a short amount of time.” (Morgan)

#### Theme 2: trapped in a cycle of fatigue

**Fig. 2:**
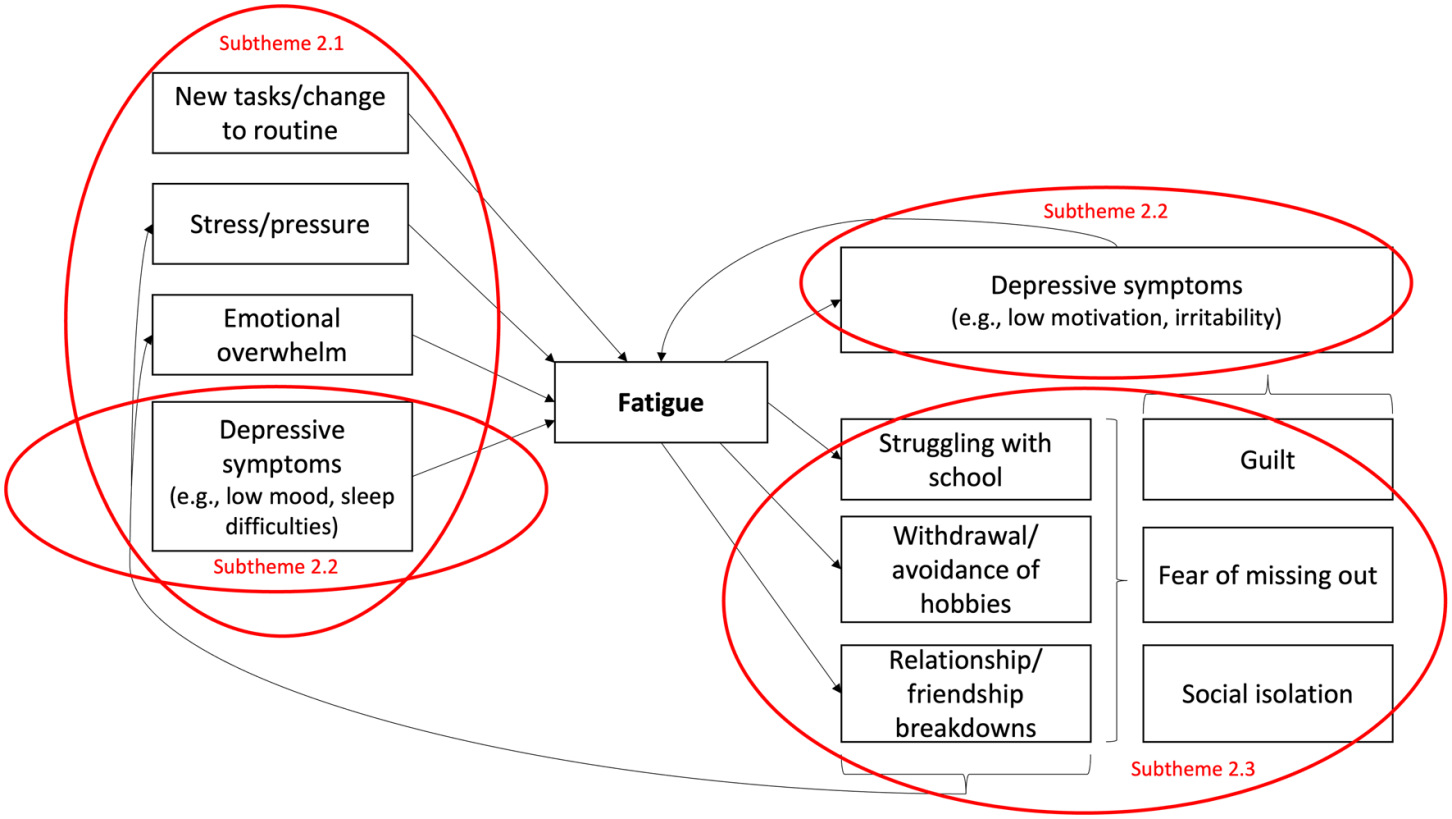
Cycle of fatigue described in Theme 2

Central to adolescents’ experiences was the feeling of being trapped in a cycle, with fatigue as a central component. This cycle is illustrated in Fig. 2 and explored through four subthemes.

##### “Everything would contribute to it”: all roads lead to fatigue

Participants highlighted a multitude of ways in which fatigue could be triggered. For some adolescents, something as innocuous as an unexpected task or argument could lead to exhaustion; for others, fatigue resulted from feelings of overwhelm or prolonged exposure to stress. This often came in the context of school.

“Me and my mum weren’t getting along very well and I felt a bit lonely and I was trying to do a lot at school like a lot of extracurricular and everything just built up and built up and I just became extremely tired, mentally and physically.” (Zainab)

Other depressive symptoms were also frequently cited as causes of fatigue, particularly low mood, struggling with motivation, sleep difficulties, and suicidal thoughts:

“[Low mood] can make my sense of fatigue even worse than it already is. Erm like when I dip, everything just turns from positive to negative.” (Freya)

Similarly, just as depressive symptoms triggered fatigue, fatigue was perceived to worsen depressive symptoms. Many symptoms were endorsed by adolescents, but low mood, low motivation, irritability, difficulty concentrating, and excessive guilt were the most common.

“I’d feel quite hopeless ‘cause I was too tired to find the motivation like I didn’t have it already but then like when I’d try and find it I couldn’t ‘cause I was so exhausted, I just didn’t have the energy.” (Megan)

The real-world impact of this for adolescents was a domino effect; with many symptoms leading into one another, it created a *“loop”* (Alfie) which adolescents found difficult to break out of.

“They build off each other, so if you don’t have the energy to do something and then your brain is also telling you that there’s no point […] why bother trying in the first place? Which can then lead you to doing nothing for ages.” (Saoirse)

##### “They’re all intertwined and linked”: difficulty disentangling fatigue from other depressive symptoms

Core to adolescents’ experience of this cycle was how hard it was to disentangle fatigue from other symptoms of depression. This was due to the co-occurrence of symptoms, which subsequently had a reinforcing effect, as described by Matty: *“They often feed into each other, it’s not – I don’t think I could ever really completely separate them”*. This was further illustrated when participants struggled to articulate the feeling of fatigue, often using other symptoms of depression as descriptors:

Interviewer: *If you were explaining fatigue to a friend, what would you say to them?*

Alfie: *It would just kind of mean just the – the just the lack of energy I guess? Just low motivation.*

##### “I haven’t got the resources in my brain to do that”: no energy to engage

Participants described how fatigue sapped their energy, leaving them with a minimal pool of resources from which to draw from. Adolescents subsequently found it difficult to maintain engagement in day-to-day activities, as described by Fran: *“I just feel like I have no-nothing left in me to like now do anything”.*

School was particularly difficult, with adolescents describing how fatigue made it hard to concentrate in class; *“I pay less attention ‘cause I’m just focusing on getting to the end of the day”* (Ellis). This difficulty concentrating had a knock-on impact on homework completion and grades: *“As I feel more tired, I do a lot less work for school and I also don’t do as well”* (Keira). For others the impact was even more extreme, leading to diminished enjoyment, low motivation, and subsequent non-attendance: *“I actually ended up skipping-skipping half the year because I was just so tired and I couldn’t bring myself to go in”* (Freya).

Challenges with remaining focused and engaged also extended to other necessary activities, such as therapy for depression:

“When I was too tired I just wouldn’t engage, I’d just sit there and give blunt answers and try and get out of there as fast as I could ‘cause I didn’t want to, I wanted to be at home in bed.” (Megan)

Even engagement with leisurely activities, such as hobbies and meeting with friends, proved a struggle. Some adolescents expressed a desire to engage with these activities but an inability to do so, whereas others described how exhaustion turned anything enjoyable into *“a chore”* (Jude), leading to complete avoidance and subsequent isolation.

“It goes to the extreme in the sense that when I am feeling tired I just don’t […] do the hobbies at all, like I just completely cut away from them and try to avoid them.” (Oskar)

“All my relationships declined ‘cause I’d just be very crabby and snappy erm and I never hung out with my friends ‘cause I was too tired so I wouldn’t go out.” (Megan)

Due to a lack of resources and diminished ability to engage, adolescents felt the need to pick and choose which activities they participated in. In this way, fatigue was understood as something that forced compromise, not allowing adolescents to fully participate in their lives, which was experienced as distressing and isolating.

“I was like so tired I would like not wanna […] go out at all 'cause I felt like I'm already going out and talking a lot at school, when I come back home […] and when I did have a break I even didn't wanna like communicate like with my family […] I like isolated myself.” (Imani)

Although compromising enabled some engagement in their activities, because it was not to the full extent, adolescents experienced feelings of guilt and a fear of missing out.

“Because I just don’t have the energy, I physically just can’t go and do it which sucks and then I feel guilty.” (Saoirse)

##### “I didn’t […] push myself to do things I knew I couldn’t do”: disrupting the cycle: protective factors

Adolescents discussed ways in which the cycle of fatigue could be broken. Distraction was the most common coping technique, usually involving low-effort activities which were within the limits of adolescents’ current energy levels. Sometimes these activities were more active, like cooking, whereas others were more passive, like listening to music; “*I’d do things within the range of which I knew I was capable of and also er a lot of escapism um like watching movies, reading books, listening to music and podcasts”* (Saoirse). These activities had the impact of allowing adolescents to *“distract my mind from being tired”* (Freya) and *“forget about my tiredness”* (Amina).

Prioritising their health, such as keeping hydrated, eating healthily, and exercising, was also perceived as helpful. Whilst adolescents acknowledged that exercising might seem contradictory in the context of fatigue, they found it allowed them to focus and could have an energising effect, reminiscent of the separation between mental and physical fatigue discussed in subtheme "“Even if I haven’t done something physical, it’s as if I have”: experienced mentally, manifests physically”.

“I do exercise and it sounds crazy like I still exercise when I’m tired, I do and it – it does help actually […] it makes me feel better and I do feel tired after it but that’s in a way where like I’ve just done exercise, of course I’m going to feel tired.” (Zainab)

#### Theme 3: stigma as a barrier to help-seeking

Despite the clear impact that fatigue had on adolescents’ lives, participants were reluctant to seek help. This seemed related to perceived stigma, both internally in the form of self-blame, and externally from a lack of understanding from others.

##### “It’s just not really an excuse to be lazy, yet I still am”: blaming self for fatigue

When discussing their experiences, adolescents often exhibited self-blame, perceiving their fatigue as reflective of an inherent flaw. This perception resulted in feelings of frustration and disappointment, which adolescents directed inwardly towards themselves; *“I was disappointed that I'd let myself down”* (Freya).

“It’s like, oh why am I tired? Like I should be—I should have enough energy to carry on doing this, I should have enough energy to work for four hours straight.” (Imani)

Adolescents felt that they should have been able to push through their tiredness, achieving the same level of productivity as their non-fatigued peers. When they were not able to do this, they understood this as laziness, and any activity that did not contribute towards productivity was viewed as *“a waste of time”* (Fran), even if it helped them to cope with their fatigue. Some adolescents recognised that this was an unfair assessment, but they were in the minority.

##### “When I do see my family it’s like, ‘oh, look who’s alive’”: lack of understanding from others

This sense of blame was not just internal, as adolescents described the blame and lack of understanding they experienced from friends and family as a result of their fatigue.

“Normally I’ll say to people that I feel tired and they’ll be like “oh just go to sleep” like they won’t (pause) wait and let me explain that it’s not something that just going to sleep can help […] it frustrates me and annoys me quite a bit because it’s just always an issue for me but I feel like I can’t talk about it.” (Morgan)

Adolescents felt that this lack of understanding stemmed from the fact that their fatigue was related to depression and did not have an identifiable physical cause. Without tangible evidence of an illness, friends and family struggled to understand where this tiredness came from, and why it seemed to have such a significant impact, leading to it not being taken seriously.

“If you have like a physical thing people’ll understand why you’re tired after you’ve broke your arm ‘cause your body wants to repair itself, but people didn’t really understand why I was tired because I was just depressed.” (Megan)

Adolescents seemed to internalise this thought process, and were subsequently reluctant to seek help, as it would be *“pointless”* (Fran). This was particularly the case in the context of seeking help from healthcare professionals; adolescents believed that that, in relation to other conditions healthcare professionals might be presented with, fatigue was not a serious enough concern and therefore not a priority.

“I just don’t think it’s a—a valid enough point to bring up I mean it’s not you know it’s not serious enough I’d—I’d say to bring up with something like that and to waste uh the person’s time on something like this.” (Oskar)

## Discussion

Findings from the current study indicate that adolescents experience fatigue as a common but complex phenomenon, one that is dynamic, on a continuum of normality, and multifaceted with biopsychosocial components. Adolescents feel trapped within a cycle of fatigue, with other symptoms of depression both contributing to and being caused by fatigue, making it difficult to disentangle one from the other. Although fatigue has a significant impact on adolescents’ lives and interferes with their ability to participate in everyday activities to the same extent as their peers, adolescents are hesitant to seek help due to internalised self-blame, a lack of understanding from others, and the subsequent perception that fatigue is not a serious enough concern to seek help for. Thus, adolescents remain trapped within the cycle of fatigue.

Adolescents understood fatigue to be on a continuum, with tiredness at one end of the spectrum and fatigue at the extreme end. This understanding moves away from historic conceptualisations as fatigue as something static and dualistic (i.e., normal versus pathological, present versus absent [45]) towards something dynamic and subjective, and is consistent with wider literature exploring adolescents’ experiences of fatigue within different conditions, such as chronic fatigue syndrome/myalgic encephalomyelitis (CFS/ME) [46] and multiple sclerosis (MS) [47]. Adolescents also distinguished between two types of fatigue: physical fatigue, and mental fatigue. Consistent with Shen et al.’s [45] definitions of physiological and psychological fatigue, adolescents understood physical fatigue to be a consequence of energy depletion or sleep disturbances, whereas mental fatigue related more to depression, reduced motivation, and intense emotional experiences. A previous study exploring young people’s experiences of depression also identified this distinction in fatigue [32]; the current study extends these findings to suggest that although adolescents experienced both types of fatigue, mental fatigue was perceived as harder to identify, harder to treat, and more greatly associated with functional impairment. In contrast, physical fatigue was perceived to be fixed through restorative activities like sleep. Whilst the multidimensionality of fatigue is a recurring topic across the wider literature (e.g., [48, 49]) and has been incorporated in common self-report measures for fatigue (e.g., CFQ [39]), the current study provides insight into how depressed adolescents conceptualise fatigue and highlights the importance of considering fatigue as more than a purely somatic symptom of depression [50]. This poses important questions regarding diagnosis and measurement, such as whether the DSM-5 and depression self-report measures commonly used in routine services adequately capture both the physical and mental components and consequences of fatigue. Clinically, this highlights issues regarding terminology and how to discuss fatigue with adolescents, alongside how to treat it. Whilst current guidelines from the National Institute for Health and Care Excellence (NICE) [51] recommend sleep hygiene as part of routine treatment, our findings indicate that focusing solely on sleep may only address the physical aspect of fatigue, leaving mental fatigue untreated. However, this seems to be the typical approach that clinicians take when presented with fatigue [35]. Further research is required to establish how these different manifestations should be addressed.

When discussing their experiences, adolescents described feeling caught in a cycle of fatigue, with symptoms of depression viewed as a trigger as well as an outcome. Adolescents subsequently found it difficult to isolate fatigue from their broader experience of depression, as many symptoms were experienced alongside fatigue as well as being reinforced by it. This is reminiscent of qualitative findings in the context of depression in adolescents CFS/ME, who also described the interplay between symptoms of CFS/ME (i.e., fatigue, cognitive impairment) and symptoms of depression (i.e., low mood, negative cognitions) as a “vicious cycle” (pg. 331) [52]. These findings support previous literature regarding the co-occurrence between fatigue and other depressive symptoms [16, 49], as well as a network analysis approach to depression, where MDD is understood to be an interconnected network comprised of co-occurring symptoms and their tendency to reinforce each other [53]. A recent systematic review of network analyses of MDD found fatigue to be the most commonly reported symptom with the highest strength centrality, suggesting it may be an important symptom in the prevention and treatment of MDD [54]. Further research is needed within the context of adolescent depression to establish if fatigue is a consistently central symptom within this population, with consequences for fatigue as a target for clinical interventions. An avenue to explore this may be through embodied cognition and interoception in mood disorders [55, 56].

Supporting previous qualitative literature in adolescent depression (e.g., [32, 34, 57]), we identified that fatigue had a substantial impact on adolescents’ psychosocial functioning and ability to participate in activities typical of their developmental stage [14, 57]. This was particularly an issue in relation to school, complimenting findings from Carroll et al. [47] who found that for adolescents with MS, fatigue-related impairments in memory and concentration affected their ability to engage in class. Extending this, we also explored the implications of such functional impairment, and identified that adolescents felt forced to compromise on which activities they participated in, and subsequently experienced feelings of isolation, guilt, and missing out, all of which have been associated with depression symptomology and severity [58–60]. Participants did, however, mention some protective factors against fatigue, which align with alleviating factors identified in a systematic review of children and adolescents’ experiences of cancer-related fatigue [61].

Findings relating to functional impairment and difficulties engaging in activities bring into question the efficacy of current depression treatments. Re-engagement with valued, pleasurable activities is often a component of psychological treatments for depression, such as CBT and Behavioural Activation (BA) [28, 51], to address low mood. However, if fatigue makes engagement with activities difficult, and forces adolescents to choose between completing required tasks, like schoolwork, and leisurely activities, like hobbies, this poses complications. From the perspective of intervention development and delivery, we need to begin considering the potential impact of fatigue, both in relation to engagement with the therapy itself, as outlined by Herring et al.’s paper on the impact of fatigue on CBT for depressed adolescents [29] and reinforced by the current findings, but also in the tasks that are set to target or alleviate other depressive symptoms, like low mood and anhedonia [28].

Despite the impact on their lives, adolescents were reluctant to seek help for fatigue, and this seemed to be a product of a lack of understanding of fatigue generally and of perceived and experienced stigma. A recent systematic review exploring barriers to adolescents seeking mental health support identified that individual-level factors, such as not viewing their problem as serious enough to require help, alongside social factors, such as perceived stigma and feared negative reactions from support networks, were both barriers to help-seeking [62]. These findings are reflected in the current study and highlight the ongoing need for increased depression literacy—the ability to recognise, manage and prevent depression and its associated symptoms—among adolescents [63] and parents of depressed adolescents [64].

However, adolescents’ perception that fatigue may not be taken seriously by healthcare professionals is not necessarily unsubstantiated. In interviews with CAMHS clinicians, it was identified that whilst fatigue was recognised as a common problem for their patients, it was not seen by clinicians as a priority for treatment. In fact, if fatigue was not raised during the initial assessment, it was unlikely to be discussed again [35]. This, combined with the subjectivity of fatigue and findings that fatigue may be difficult to distinguish or disentangle from other symptoms of depression, reinforces the need to continue advancing our understanding of symptoms like fatigue, and clinicians’ confidence in identifying and addressing it.

### Strengths and limitations

A strength of this study is in the clinical diversity of the sample. As we recruited from clinical and community settings, we have representation from adolescents with current, historic, and no experience of engagement with CAMHS. This is important, as many studies exploring adolescent depression focus exclusively on clinical populations, despite knowledge that many adolescents struggling with depression do not have access to these services [37]. We also managed to capture the perspectives of adolescents with a range of depression severity, in part due to the focus on elevated symptoms rather than clinical diagnosis; however, because we did not consider MDD diagnosis, it is possible that the most severe cases of fatigue and depression were not captured.

The sample was not demographically diverse, comprised predominantly of adolescents identifying as White British and cisgender, although there was some gender diversity. Despite the study recruiting for adolescents aged 11–18 years old, most of the sample were older adolescents, meaning the perspectives of those aged 11–13 years old is missing. Considering research suggests that fatigue is associated with increasing age [65] this may not be surprising; however, it would still be interesting for future qualitative research to explore younger adolescents’ experiences of fatigue and identify any similarities or differences between groups. Nevertheless, as this was an exploratory study, we did not seek to recruit a representative sample and do not assume that our findings will generalise to a wider population of depressed adolescents.

Finally, a Young Person’s Advisory Group, comprised of young people with current or previous experience of depression, was consulted at every stage of this study, from design to dissemination. This enhanced the reflexivity of the study and strengthened both the interpretation of the data and applicability of the results.

### Implications for clinical practice

Fatigue is a common and debilitating symptom of adolescent depression but is often overlooked. The current study highlights that adolescents may be reluctant to seek help for fatigue despite its significant impact, indicating a need to ensure that fatigue is explicitly discussed during assessment. As adolescents may not commonly use the term ‘fatigue’ or may find it difficult to disentangle from other depressive symptoms, using different terminology (e.g., ‘tired’, ‘exhausted’, ‘low energy’) and asking about fatigue in the context of other symptoms, such as low motivation, may be helpful. Within this also needs to be consideration of the distinction between mental and physical fatigue. Clinicians may consider using structured questionnaires like the CFQ to aid their assessment of fatigue.

It is also important to consider fatigue in the context of treatment. Firstly, fatigue may interfere with adolescents’ ability to engage in evidence-based psychological therapies like CBT and BA, given its impact on concentration and motivation. This means that adaptations may be required, such as reduced session length and flexibility with between session tasks [29]. Secondly, in some instances where fatigue is central to the maintenance formulation, there may be a need to address it more directly [66]. This could be achieved through implementing interventions designed with the intention of treating fatigue, or by integrating fatigue-specific content into existing treatments for depression. Ideas may be taken from a recent systematic review of nonpharmacological interventions for fatigue in adolescents, which identified that psychoeducation, CBT and physical activity all offered some degree of promise in addressing adolescent fatigue, utilising techniques such as goal setting and gradually increasing activity [67]. As depressed adolescents can be particularly critical of themselves, with a tendency to self-blame, an important part of this work may be helping them to develop a more compassionate approach towards allowing themselves to modulate their initial expectations, set a baseline of activity, and gradually increase what they are able to do.

## Conclusion

We found that adolescents with depression experience fatigue as a complex, dynamic symptom that is intertwined with other depressive symptoms and leaves them feeling trapped in a never-ending cycle. Fatigue impacts adolescents’ ability to participate in everyday activities, forcing them to compromise in what they engage in, and leading to feelings of isolation and missing out. Despite the significant impact, adolescents do not feel able to seek help for fatigue due to internal and external experiences of stigma, and the perception that fatigue is not serious enough for support. Further research is needed to establish whether current psychological treatments for depression effectively address fatigue in depressed adolescents, and if not, what adaptations may need to be implemented.

### Supplementary Information

Below is the link to the electronic supplementary material.

Supplementary file1 (DOCX 94 KB)

## Data Availability

Anonymised data from the current study are available from the corresponding author, NH-S, upon reasonable request.
