# Supplementary material for: “I’m always going to be tired”: a qualitative exploration of adolescents’ experiences of fatigue in depression
Source: Eur Child Adolesc Psychiatry. 2023 Jun 10;33(5):1369–81. doi: 10.1007/s00787-023-02243-3 (PMC10257178; doi:10.1007/s00787-023-02243-3)
Supplement: Supplementary file 1 — Supplementary file1 (DOCX 94 KB) [file 787_2023_2243_MOESM1_ESM.docx]

Online Supplementary Materials

**Topic Guide**

**INTRODUCTION AND REMINDERS**

***Introduce self and study:***

- My name is Nina, PhD student at the University of Bath, research into child and adolescent mental health

***Check consent and understanding:***

- Before I say anything else, I just want to check your understanding of the study
- Do you feel like you have to take part in this study? **Correct answer: No**
- Do you know what you are being asked to do today? **Correct answer: Discuss experiences of depression and/or tiredness**
- What can you do if you no longer want to take part? **Correct answer: Withdraw (at any time, without giving a reason)**

***Reminders:***

- Study purpose – learning about experiences of depression and other symptoms
- Taking part is voluntary and will not affect any care you receive
- You also have the right to withdraw at any time during the interview, without giving a reason
- After the interview, you can withdraw again without giving a reason, up to 15 days afterwards
- You can also ask to take a break whenever you want, and we can also stop or reschedule the interview
- You don’t have to answer any questions you don’t want to, and there are no correct answers – I’m interested in hearing your honest thoughts
- I will also be recording the interview today – this recording will be kept securely, only the research team will have access; I will transcribe the data (explain) and during this will removal any identifiable information, e.g. name

Complete safety plan agreement form (read confidentiality info from sheet)

Before we start, do you have any questions?

***Start recording!***

**ICE-BREAKER**

General prompts

What makes you say that?

Can you tell me more about this?

Can you explain what you mean by ‘X’?

Can you give me an example of what you mean?

In what sense? / What do you mean?

How does that feel? / How did you find that?

What did you do?

What were you thinking at that time?

1. To start, can you tell me how you found out about this study and what interested you about it?

**KNOWLEDGE, UNDERSTANDING AND DESCRIPTIONS OF FATIGUE**

1. Now or in the past, has there ever been a time when you’ve felt like you have no energy or just felt really tired? IF YES TO QUESTION 2, CONTINUE; IF NO, SKIP TO PAGE 5
2. What words would you use to describe this feeling?
   - *Can you explain what you mean by ‘X’?*
3. Have you heard the term ‘fatigue’ before?
   - **If yes:**
     1. *What does ‘fatigue’ mean to you?*
     2. *If you were explaining ‘fatigue’ to a friend, how would you define it?*
     3. *Would you use ‘fatigue’ as a word to describe what we spoke about just now, that lack of energy? Can you explain your answer to me?*
   - **If no:**
     1. *What does hearing the word ‘fatigue’ make you think of?*
     2. *Would you use ‘fatigue’ as a word to describe what we spoke about just now, that lack of energy? Can you explain why/why not?*
4. How could you tell if someone else your age was feeling fatigued or tired?
   - *Can you explain your answer to me?*
   - *You mentioned X. Can you explain why you’d recognise this as tiredness?*

**PERSONAL EXPERIENCES OF FATIGUE**

1. Can you tell me about a particular point in time when you felt really tired/fatigued?
   - *Can you talk me through what happened? E.g. how did it start, how did you feel, what did you do*
   - *Was there anything that triggered this? If so, what was it?*
   - *What did other people say or do when you felt like this?/How did they react?*
   - *What did you do to help you cope?*
2. When you feel tired...
   - *What helps you feel less tired?*
   - *What makes your tiredness worse?*
   - *What things do you do regularly to help you cope?*
   - *How does tiredness impact your life, if at all? E.g., family/friends, school/work, hobbies/activities*
   - *Can you give me an example of a time your tiredness impacted X?*
   - *How did this make you feel?*

General prompts

What makes you say that?

Can you tell me more about this?

Can you explain what you mean by ‘X’?

Can you give me an example of what you mean?

In what sense? / What do you mean?

How does that feel? / How did you find that?

What did you do?

What were you thinking at that time?

1. Have you spoken with other people about these feelings of tiredness?
   - **If yes:**
     1. *Who did you talk to?*
     2. *What made you decide to talk to them?*
     3. *Can you talk me through what happened when you told them?*
     4. *How did you explain your tiredness to them?*
     5. *How did they respond?*
     6. *How did their response make you feel?*
   - **If no:**
     1. *Have you thought about talking to someone? If so, who?*
     2. *Is there any particular reason you haven’t?*
     3. *What would help you to reach out?*

**PERSONAL EXPERIENCES OF DEPRESSION AND INVOLVEMENT OF FATIGUE**

1. Now I’d like to focus on your experiences of depression more broadly. What does depression feel like for you?
   - *Can you talk me through a recent time when you felt really depressed? What was going on for you?*
   - *Can you tell me more about some of the symptoms you experience?*
   - *How do you cope with these symptoms/feelings?*
   - *What is the impact of X symptom on your life?*
2. Now I want to bring tiredness back into the picture. How do you think tiredness relates to or interacts with these other feelings/symptoms, if at all?
   - *Can you explain your answer to me?*
   - **Low mood/Sleep disturbances/Anhedonia/Difficulty concentrating:**
     - *Can you tell me more about what it feels like to feel X and tired?*
     - *Are there any other symptoms you often experience alongside X and tiredness?*
       - *What are these? Can you tell me more about them? How do you think they affect each other?*
     - *Do you think there is a difference between X and tiredness?*
       - **If yes:** *How do you distinguish between X and tiredness? / What’s the difference?*
       - **If no:** *Can you explain your answer to me? / Why would you say they’re the same?*
3. We’re almost at the end of the interview. Thinking about all the symptoms of depression you experience, where would you rank tiredness?
   - *High? Low? In the middle?*
   - *Can you explain your answer to me?*
   - *What symptoms would you rank above/below tired? Why?*

**END OF INTERVIEW AND DEBRIEF**

1. How did you find answering these questions?
2. Before I stop the recording, is there anything else you would like to tell me?

- ***Stop recording!***
- Thank participant and remind of reimbursement; choose which option.
- Reiterate confidentiality and right to withdraw; within 15 days of today.
- Would you like to receive an update about the study once I’ve written up the findings?
  - If yes, get verbal consent for email address to be used for this purpose.
- Will email a debrief sheet within 24 hours which has a list of sources of support; these sources are also on the participant information sheet. Can also email with questions.
- Reminder to contact CAMHS clinician or GP if distressed/wanting to talk further about some things we may have discussed.

IF NO TO QUESTION 2

**FATIGUE IN DEPRESSION**

1. You’ve said that you have never had a time when you’ve felt like you had no energy or felt really tired. Has there been a time when you’ve felt a bit like that, or the closest way to that?

- **If yes:**
  1. *Could you tell me about that time? What was happening?*
  2. *How would you best describe how you felt at that time?/What words would you use to describe your feelings at that time?*
  3. *When you were feeling like this, did you also feel sad or low?*
  4. **If yes:**
     - *Why do you think you might feel these emotions at the same time?*
     - *Does this feel different than when you’re feeling tired on its own? Can you explain your answer to me?*
     - *Is there a difference between feeling low and feeling tired? If so, what do you think this difference is?*
  5. **If no:**
     - *Are there any other symptoms you think you also experience alongside feeling low and feeling tired? What are these? Can you tell me more about them? How do you think they affect each other?*
     - *How do you tell tiredness apart from these other symptoms?*
- **If no:** move on

**PERSONAL EXPERIENCES OF DEPRESSION AND OTHER SYMPTOMS**

1. I’m really interested in hearing more about your experiences of feeling sad or low. Can you tell me about a particular point in time when this happened?
   - *Can you talk me through what happened? E.g. how did it start, how did you feel, what did you do*
   - *Was there anything that triggered this? If so, what was it?*
   - *What did other people say or do when you felt like this?/How did they react?*
   - *What did you do to help you cope?*
2. When you feel low...
   - *What helps to improve your mood?*
   - *What makes your mood worse?*
   - *What things do you do regularly to help you cope?*
   - *What is the impact of feeling low or depressed on your life? E.g.,*
     1. *Family and friends?*
     2. *School/work?*
     3. *Hobbies and activities?*
   - *Can you give me an example of a time your low mood impacted X?*
   - *How did this make you feel?*
3. Have you spoken with other people about your low mood?
   - **If yes:**
     1. *Who did you talk to?*
     2. *What made you decide to talk to them?*
     3. *Can you talk me through what happened when you told them?*
     4. *How did you explain your low mood to them?*
     5. *How did they respond?*
     6. *How did their response make you feel?*
   - **If no:**
     1. *Have you thought about talking to someone? If so, who?*
     2. *Is there any particular reason you haven’t?*
     3. *What would help you to reach out?*
4. When you are feeling low or sad, are there any other feelings or thoughts that you have a lot?

- *Can you tell me a bit more about these?*
- *How do these make you feel? / Can you describe what it felt like?*
- *Was there anything that caused/triggered this?*
- *How long did these feelings/thoughts last for?*

1. How do these other feelings or thoughts impact your day-to-day life?

- *Can you describe what this looks like for me?*
- *What helps you cope with this?*
- *Is there anything that makes these feelings/thoughts worse? If so, how did you deal with it?*

**END OF INTERVIEW AND DEBRIEF**

1. How did you find answering these questions?
2. Before I stop the recording, is there anything else you would like to tell me?

- ***Stop recording!***
- Thank participant and remind of reimbursement; choose which option.
- Reiterate confidentiality and right to withdraw; within 15 days of today.
- Would you like to receive an update about the study once I’ve written up the findings?
  - If yes, get verbal consent for email address to be used for this purpose.
- Will email a debrief sheet within 24 hours which has a list of sources of support; these sources are also on the participant information sheet. Can also email with questions.
- Reminder to contact CAMHS clinician or GP if distressed/wanting to talk further about some things we may have discussed.

**Reflexivity Statement**

The lead researcher is a mixed heritage woman currently conducting her PhD at the University of Bath on the topic of fatigue within adolescent depression. She was diagnosed with Major Depressive Disorder at 14 years old and received treatment from Child and Adolescent Mental Health Services (CAMHS) until she was discharged at the age of 18. The lead researcher was supported throughout the project by a team of three supervisors, all of whom are clinical psychologists and academics with a background in child and adolescent mental health. All supervisors had experience of conducting and supervising projects related to adolescent depression, with two also previously having worked within CAMHS, and one having previous experience of working within a specialist paediatric Chronic Fatigue Syndrome/Myalgic Encephalomyelitis service.

For the duration of the project, the supervisory team frequently met with the lead researcher, both individually and as a group, to discuss and reflect on how their experiences, values, beliefs, and identities influenced how the project was approached and conducted. To practice and maintain reflexivity between supervisory meetings, the lead researcher kept a reflexive diary, and regularly attended research group meetings with other academics also researching depression in young people. A Young Person’s Advisory Group, created by the lead researcher and one of the project supervisors as part of this research group, was also consulted throughout the project. The group comprised up to 7 young people with current or historic experience of depression, who provided valuable input regarding the development of the study materials and each iteration of the analysis.
